# Immunosuppressant prescription pattern and trend in kidney transplantation: A multicenter study in Korea

**DOI:** 10.1371/journal.pone.0183826

**Published:** 2017-08-28

**Authors:** Ji-Yeun Chang, Jihyun Yu, Byung Ha Chung, Jaeseok Yang, Sung-Joo Kim, Chan-Duck Kim, Sang-Ho Lee, Jong Soo Lee, Joong Kyung Kim, Cheol Woong Jung, Chang Kwon Oh, Chul Woo Yang

**Affiliations:** 1 Division of Nephrology, Department of Internal Medicine, Seoul St. Mary’s Hospital, College of Medicine, The Catholic University of Korea, Seoul, Republic of Korea; 2 Transplantation Center, Seoul National University Hospital, Seoul, Republic of Korea; 3 Department of Surgery, Samsung Medical Center, Sungkyunkwan University School of Medicine, Seoul, Republic of Korea; 4 Division of Nephrology, Department of Internal Medicine, Kyungpook National University Hospital, Daegu, Republic of Korea; 5 Division of Nephrology, Department of Internal Medicine, Kyung Hee University Hospital at Gangdong, Seoul, Republic of Korea; 6 Division of Nephrology, Department of Internal Medicine, Ulsan University Hospital, Ulsan, Republic of Korea; 7 Division of Nephrology, Department of Internal Medicine, Bong Seng Memorial Hospital, Busan, Republic of Korea; 8 Department of Surgery, Korea University Anam Hospital, Seoul, Republic of Korea; 9 Department of Surgery, Ajou University Hospital, Suwon, Republic of Korea; Istituto Di Ricerche Farmacologiche Mario Negri, ITALY

## Abstract

**Background:**

The actual prescription pattern of immunosuppressive agents in kidney transplantation is unclear.

**Methods:**

We investigated the pattern and trend of immunosuppressive treatment for kidney transplant patients in South Korea. A total of 636 patients at nine transplant centers were enrolled and followed for one year. We reviewed medical records and evaluated induction therapy, as well as the changing pattern and cause of maintenance therapy.

**Results:**

Most patients (n = 621, 97.6%) received induction therapy often comprising basiliximab (n = 542, 85.2%). The triple therapy including calcineurin inhibitor, mycophenolic acid, and steroids was the major initial maintenance immunosuppression (n = 518, 81.4%), but its proportion decreased by 14% (81.4% to 67.5%) after 1 year. Almost 40% of patients changed immunosuppressive regimen during the 1-year follow-up, most often at an early period (60.2% within the first 4 months). The primary reason for the change was gastrointestinal discomfort (n = 113, 29.8%), followed by infection (112, 29.6%). The most common changing pattern was mycophenolic acid withdrawal (n = 155, 39.1%).

**Conclusion:**

The initial immunosuppressive regimen is prone to change within the first year of kidney transplantation. Further studies are needed to evaluate the benefits and risks in patients who changed immunosuppressants.

## Introduction

The introduction of calcineurin inhibitors (CNIs) in clinical practice much improved the short-term graft survival rates, but its effects in long-term graft longevity were not significant in kidney transplantation (KT) recipients [1]. Therefore, diverse combination of protocols were developed to improve immunosuppression with minimizing side effect of CNIs., Among them, triple therapy consisting of low-dose tacrolimus (TAC), mycophenolic acid (MPA) and steroid is popular in current practice as a maintenance ISx treatment. However, it is not well known whether the initial immunosuppressive regimens are maintained during the post-transplant periods in real-world medical settings. Therefore, we investigated practices in ISx prescription via national survey and compared the results with those of previous reports.

## Patients and methods

### Design

We conducted a multi-center retrospective, chart-review study in order to evaluate the ISx use patterns in patients who underwent KT in South Korea. Trained renal fellows or clinical research coordinators at each center collected the primary data based on identical data-selection criteria. All the investigators reviewed the dataset provided and reorganized the information for analysis according to the study purpose. The local Institutional Review Board (IRB) approved this study at each center and all clinical investigations were conducted according to the principles expressed in the Declaration of Helsinki. Informed consents were waived off owing to the retrospective nature of this study. None of the transplant donors were from a vulnerable population and all donors or next of kin provided written informed consent that was freely given.

IRB approval number: Seoul St. Mary’s Hospital, KC14RSME0215; Seoul National University Hospital, H-1403-127-572; Samsung Medical Center, 2014-04-047; Kyungpook National University Hospital, KNUH 2014-04-026; Kyung Hee University Hospital at Gangdong, KHNMC 2014-03-011; Ulsan University Hospital, UUH 2014-03-019; Korea University Anam Hospital, ED14049; Ajou University Hospital, AJIRB-MED-MDB-14-098; Bong Seng Memorial Hospital, BSIRB-2014-006.

### Study population

The study group consisted of patients aged ≥ 18 years who received KT between January 1 and December 31, 2012, at nine transplant centers in South Korea. Patients who received a multi-organ transplant, as well as patients without medical records or laboratory data one year after the KT, were excluded. Of 641 patients, retrieval data were available for 636 transplants. The 5 excluded patients had main characteristics that were similar to the included patients.

### Data collection

The primary data contained demographic information (age, gender, height, and weight), patient’s disease status information (primary renal disease, pre-transplant dialysis status, pre-transplant and post-transplant comorbidities), transplant-related immunological baseline characteristics (transplant number, donor type, T-cell/B-cell cross matching test results by both the complement-dependent cytotoxicity method and the flow cytometry method, percent panel reactive antibody test (PRA), ABO compatibility status, and HLA mismatching number based on three HLA loci (A, B, DR)), details of ISx prescription (induction therapy regimen, initial maintenance regimen type and dose, modifications of ISx with cause and date), and information on allograft function (graft failure, serum creatinine level and estimated glomerular filtration rate (eGFR) calculated using CKD-EPI formula). Serum creatinine level and eGFR were measured at discharge immediately following and one year after the KT.

### Definitions

#### Classification of maintenance immunosuppressants

Maintenance immunosuppressive agents were grouped according to the combination of drugs: 1) CNI-based triple therapy consisting of CNI (CSA or TAC), MPA (mycophenolate mofetil [MMF] or enteric-coated mycophenolate sodium [EC-MPS]), and steroid, 2) steroid-sparing regimen consisting of CNI and MPA, 3) MPA-sparing regimen consisting of CNI and steroid, 4) SRL-based regimen, which is defined as SRL with any drugs, and 5) other regimens.

#### Immunosuppressant change

The “ISx change” was defined as 1) withdrawal of a certain drug, 2) addition of a certain drug, and 3) switching drug “A” to drug “B” during the first year post-transplant. Modifications that were not considered as an ISx change included: 1) dose modification of the same drug, 2) re-administration of the same drug after a temporary stopping, and 3) change based on a typical protocol. All kinds of steroids were regarded as the same drug in this study. For example, if a patient stopped MPA due to infection and restarted after the infection treatments, we considered it as MPA withdrawal due to infection, but not MPA addition due to recovery. The temporary withdrawal of CNI or MPA during anti-thymocyte globulin (ATG) treatment was also not considered as ISx change because it was a routine ATG protocol.

#### Periods of time after the transplantation

The one year time period of this study period was evenly divided into three groups: 1–4 months, 5–8 months and 9–12 months. We defined these three divisions of the period as the first, second, and third tertile, respectively.

### Assessment of post-transplant complications

Acute rejection included both acute T-cell mediated and acute antibody-mediated rejection which were diagnosed by the pathologist at each center based on the Banff classification scheme [2]. Infection was defined as an illness resulting from any disease-causing organism. Cytomegalovirus (CMV) disease was defined as organ involvement of CMV and BK virus (BKV) infection included BK viremia greater than 10,000 copies /mL or BKV-associated nephropathy proven by allograft biopsy. GI problems included both subjective symptoms, such as abdominal bloating, dyspepsia, and pain, as well as objective sign like diarrhea or constipation. Cardiovascular events included myocardial infarction and symptomatic heart failure (NYHA class II to IV). Diabetes mellitus (DM) complications included uncontrolled blood glucose, DM foot, DM retinopathy, diabetes ketoacidosis and hyperglycemic hyperosmolar nonketotic coma. Bone marrow suppression was defined as a state with neutropenia (white blood cell count < 4000 per microliter), and/or thrombocytopenia (platelet count < 150,000 per microliter). Anemia was not included due to the obscurity of onset. Nephrotoxicity was given a presumptive definition. When kidney function was a affected without evidence of rejection, clinicians considered the ISx having nephrotoxicity. Graft failure was defined as the re-establishment of long-term dialysis treatment.

### Statistical analyses

Descriptive statistics were used in all analyses. We presented continuous data as means ± standard deviations (SD) or median with interquartile ratio, and categorical data as counts or frequencies. The Mann-Whitney U test was used to compare the nonparametric continuous data, while the Fisher’s exact test was used to compare the categorical data. All analyses were conducted by using the IBM SPSS Statistics 24 software (Armonk, NY: IBM Corp.).

## Results

### Baseline characteristics

Included patients were in mid-40s with a 3-year-duration of dialysis, and 16.7% were preemptive KT. Glomerulonephritis was a leading cause of ESRD, followed by DM. Donor type was living-donor-basis (60.4%), and most patients were non-sensitized. However, a small number of ABO incompatible, HLA-sensitized and re-transplant patients were also included (Table 1).

**Table 1:** Baseline characteristics of kidney transplant recipients at registration.

Variable		Value
Recipient age		45.4±12.7
Recipient, male, n (%)		377 (59.3)
Deceased donor, n (%)		252 (39.6)
Retransplantation, n (%)		45 (7.1)
Dialysis modality, preemptive: HD: PD		106: 442: 124
Duration of dialysis, months		36.0 (6.0–71.0)
Causes of ESRD, n (%)		
	Glomerulonephritis	226 (35.5)
	Unknown	149 (23.4)
	Diabetes mellitus	112 (17.6)
	Hypertension	87 (13.7)
	Polycystic kidney disease	37 (5.8)
	Lupus nephritis	8 (1.3)
	Reflux nephropathy	4 (0.6)
	Others	13 (2.0)
Total HLA mismatch number		3.3±1.6
Panel reactive antibody, n (%)		
	Class I > 50%	49 (7.7)
	Class II > 50%	30 (4.7)
Cross matching test positivity, n (%)		
	T-cell	6 (0.9)
	B-cell	18 (2.8)
ABO incompatibility		85 (13.4)

Continuous variables with normal distributions are presented with mean±S.D, whereas continuous variables with non-normal distributions are presented with median (interquartile range)

Abbreviations: HD, hemodialysis; PD, peritoneal dialysis; ESRD, end stage renal disease; HLA, human leukocyte antigen

### Induction therapy

Table 2 shows the characteristics and graft outcome of KT recipients with or without induction and as a type the induction regimen. Most of the patients (n = 621, 97.6%) all but 15 (2.4%) underwent induction ISx therapy. Basiliximab, an anti-CD25 antibody, was a more commonly used agent (n = 542, 85.2%) than ATG (n = 79, 12.4%). The use of ATG was greater in highly sensitized patients, deceased-donor transplants, re-transplants, and ABO incompatible transplants. ISx change was more frequent in patients with ATG induction than in patients without ATG induction (Table 2). The graft failure rate and graft function were not different with or without induction therapy, nor were they different between basiliximab or ATG induction.

**Table 2:** Characteristics according to induction therapy.

		Total Study Population			Patients with Induction Tx.		
		Induction(-)	Induction(+)	p-value	Basiliximab	ATG	p-value
		n = 15	n = 621		n = 542	n = 79	
Age		42.8±13.0	45.4±12.6	0.191	45.1±12.9	47.6±10.7	0.061
Male, n (%)		11 (73.3)	366 (58.9)	0.301	321 (59.2)	45 (57.0)	0.715
DDKT, n (%)		0 (0.0)	252 (40.6)	0.001	206 (38.0)	46 (58.2)	0.001
Retransplant, n (%)		0 (0.0)	45 (7.2)	0.616	30 (5.5)	15 (19.0)	<0.001
Highly sensitized, n (%)		1 (6.7)	77 (12.4)	0.708	53 (9.8)	24 (30.4)	<0.001
ABO incompatibility, n (%)		2 (13.3)	83 (13.5)	1.000	66 (12.3)	17 (21.5)	0.033
ISx Change, n (%)		1 (6.7)	250 (40.3)	0.007	204 (37.6)	46 (58.2)	0.001
Graft Failure, n (%)		0 (0.0)	2 (0.3)	1.000	2 (0.4)	0 (0.0)	1.000
eGFR (mL/min/1.73m^2^)							
	At KT	74.7±20.1	72.2±25.1	0.704	72.6±24.7	69.1±27.4	0.250
	1-year after KT	65.7±13.2	63.9±21.1	0.748	64.4±21.1	60.4±21.0	0.115
	∆eGFR (1 year)	-7.1 (28.2)	-8.0 (23.2)	0.960	-7.6 (23.1)	-10.2 (26.3)	0.641

Continuous variables with normal distributions are presented with mean±S.D.

Continuous variables without normal distributions are presented with median (interquartile range).

**∆**eGFR represented the slope of eGFR between the time of discharge and 1-year after the transplantation.

Abbreviation: Tx., treatment; ATG, anti-thymocyte globulin; DDKT, deceased-donor kidney transplantation; ISx, immunosuppressant; eGFR, estimated glomerular filtration rate.

### Initial and one-year maintenance therapy

The majority of KT recipients (81.4%) started their maintenance ISx with TAC-based triple therapy. All patients initially used CNI, and the proportion of TAC and CSA was 87.7% and 12.3%, respectively. Of MPA, MMF was predominant as compared with EC-MPS at the time of the KT (53.1% vs. 40.1%). On average, patients received 1.4 g of MMF and 0.8 g of EC-MPS. The dose of both MPA was higher in patients receiving TAC than in patients receiving CSA (MMF, 1439.4 mg vs. 1433.0 mg, p-value = 0.940; EC-MPS, 875.9 mg vs. 598.6 mg, p-value <0.001).

**Fig 1:**
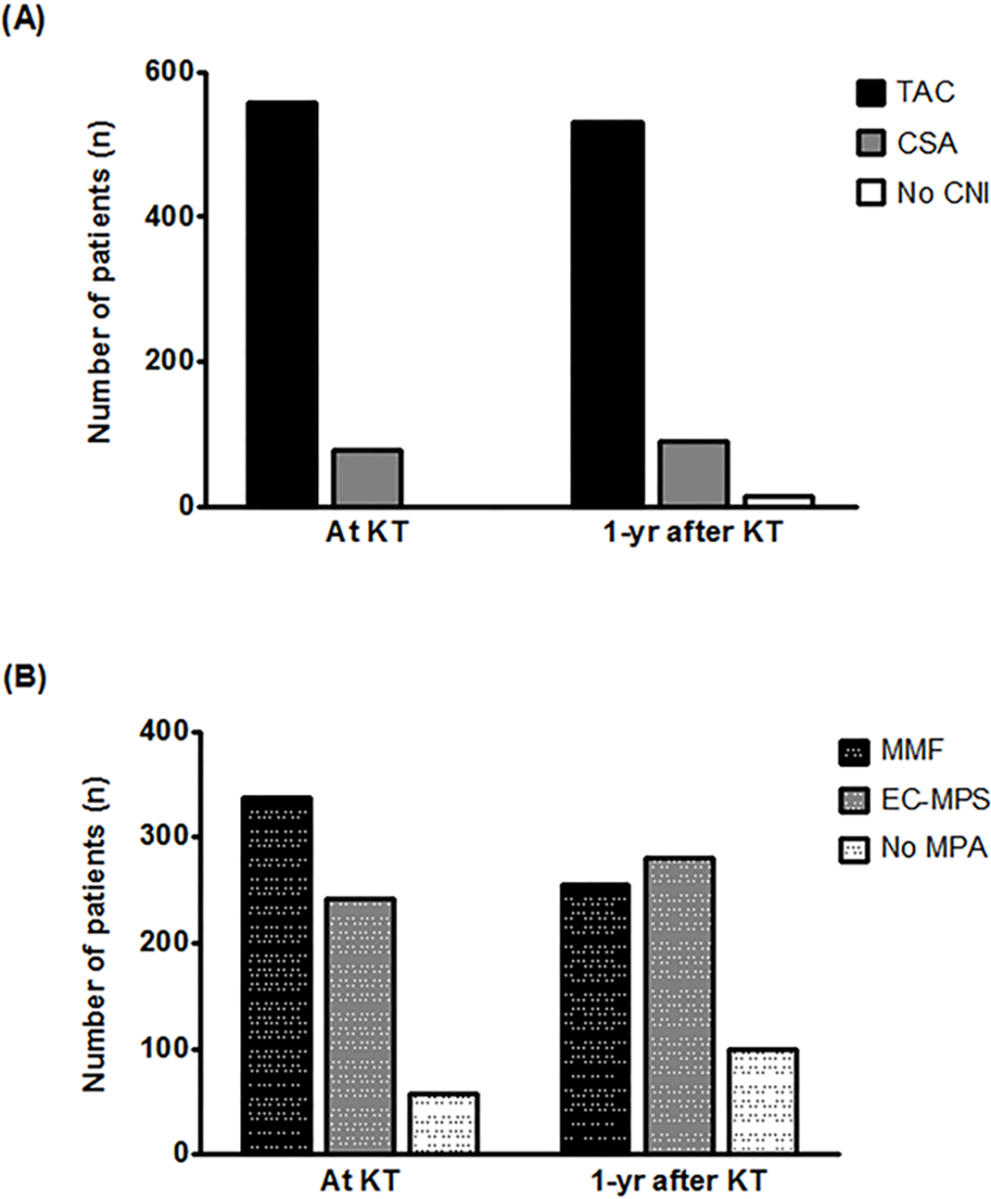
Initial and one-year CNI and MPA prescription The vertical bar graph illustrates the prescription frequency of CNI (A) and MPA (B) at discharge and one year after the transplant. In terms of CNI, TAC is far more prescribed than CSA is (At KT, 558 vs. 78; 1yr after KT, 531 vs. 91). In terms of MPA, the beginning stage prescription for MMF is more than that for EC-MPS, but after one year, EC-MPS prescription rate exceeds that of MMF (At KT, 337 vs. 255; 1yr after KT, 241 vs. 281). Abbreviation: TAC, tacrolimus; CSA, cyclosporine A; CNI, calcineurin inhibitor; KT, kidney transplantation; yr, year; MPA, mycophenolic acid; MMF, mycophenolic mofetil; EC-MPS, enteric-coated mycophenolate sodium.

At one year, CNI-based triple regimen was still the most common ISx, but its use had decreased by 14% compared to that at the time of KT (81.4 to 67.5%). On the contrary, the prescription rate of steroid-sparing (11.3 to 13.2%) and SRL-based regimens (0.3 to 7.5%) increased. The total MPA prescription rate had decreased compared with the initial rate (93.1 to 82.1%), but the absolute number of KT recipients with EC-MPS increased, thereby resulting in a reverse MMF to EC-MPS ratio (1.3 to 0.9) (Table 3, Fig 1).

**Table 3:** Maintenance immunosuppression regimen.

Group	At Discharge after the KT	One Year after the KT
1: CNI + MPA + Corticosteroid	518 (81.4)	429 (67.5)
2: CNI + MPA	72 (11.3)	84 (13.2)
3: CNI + Corticosteroid	40 (6.3)	38 (6.0)
4: srl-based	2 (0.3)	48 (7.5)
5: others	4 (0.6)	37 (5.8)

Data account for the number (%) of the patient; total number = 636.

Abbreviation: KT, kidney transplantation; CNI, calcineurin inhibitor, MPA, mycophenolic acid; SRL, sirolimus.

### Time and frequency of overall immunosuppressant changes

**Fig 2:**
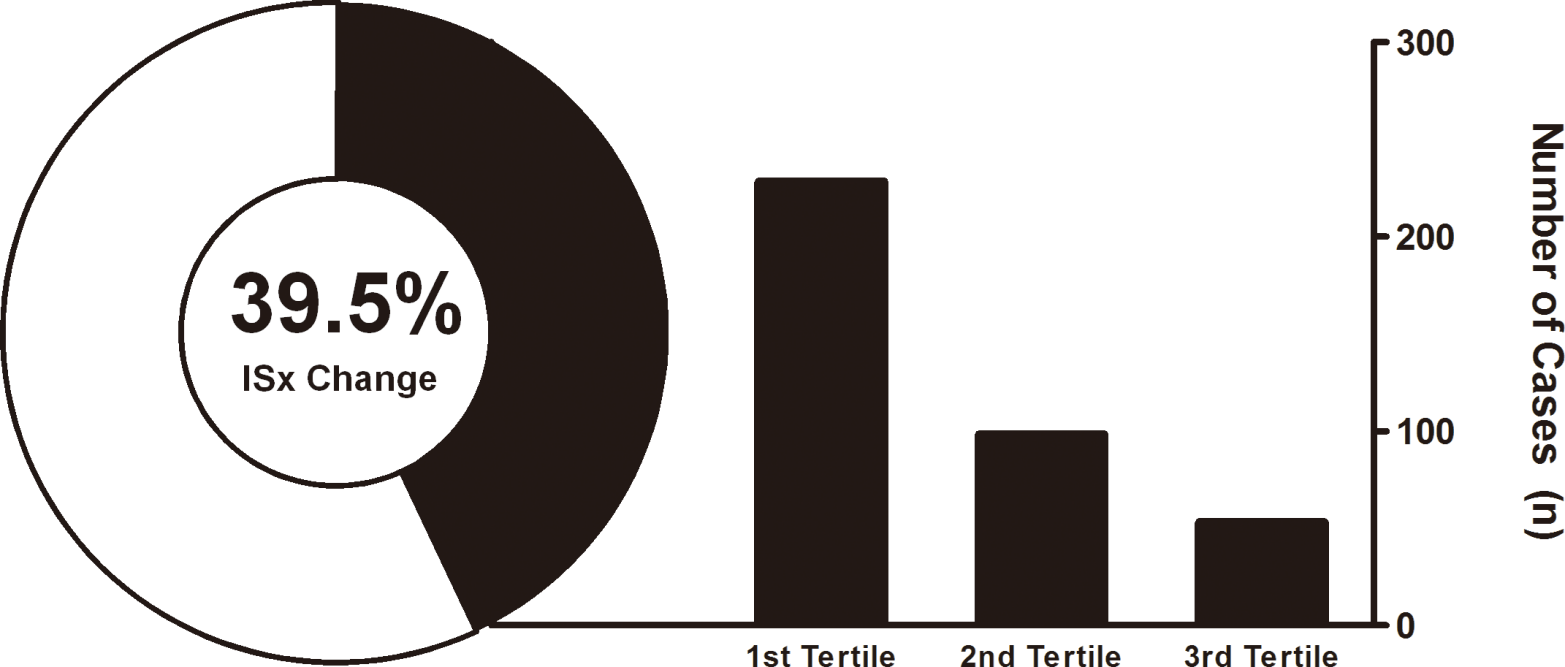
Immunosuppressive regimen change within one year. The sunburst graph on the left side represents the proportion of immunosuppression regimen changes and the bar graph on the right side represents the number of immunosuppressant changes as a function of the post-transplant period. A 39.5% of transplant recipients changed their ISx within one year of the transplant. Abbreviation: ISx, immunosuppressant.

Almost half of all patients (39.5%) changed their maintenance ISx one or more times during the first year after the KT (1.51 per person per year). Within one year, a total of 379 ISx changing cases with 396 changing patterns existed; in 16 cases, 2 to 3 types of ISx changing patterns were applied simultaneously (eg. withdrawal of both MPA and steroid). A majority of them (60.2%) occurred in the first 4 months (Fig 2).

### Cause of immunosuppressive regimen change

**Fig 3:**
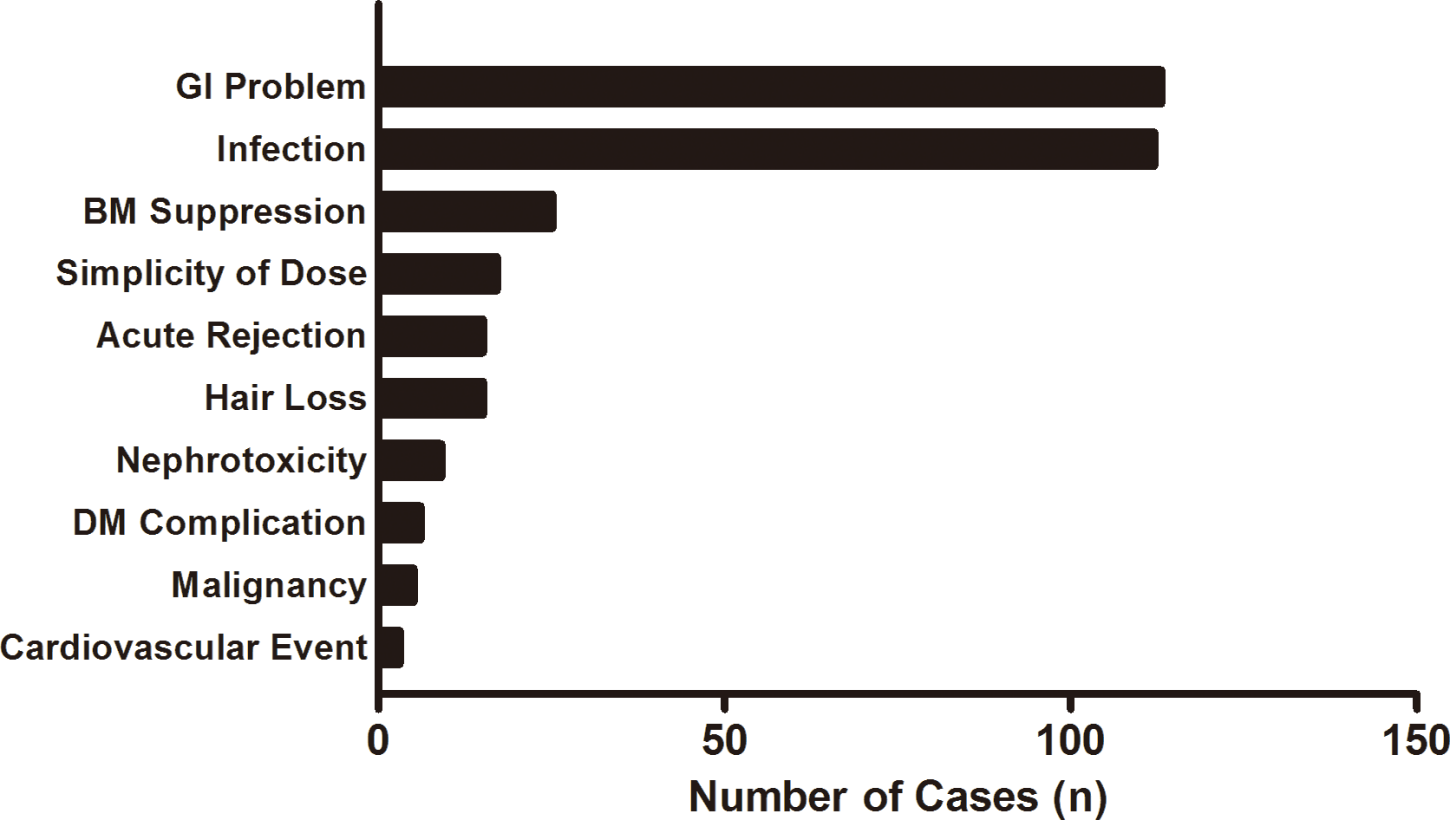
Causes of immunosuppressive regimen change. The horizontal bar graph represents the causes of ISx change in order of frequency. The total number of changing cases was 379. GI problems most commonly resulted in ISx changes (113 times, 29.8%), followed by infection (112 times, 29.6%). Abbreviation: GI, gastrointestinal; BM, bone marrow; DM, diabetes mellitus; ISx, immunosuppressant.

**Fig 4:**
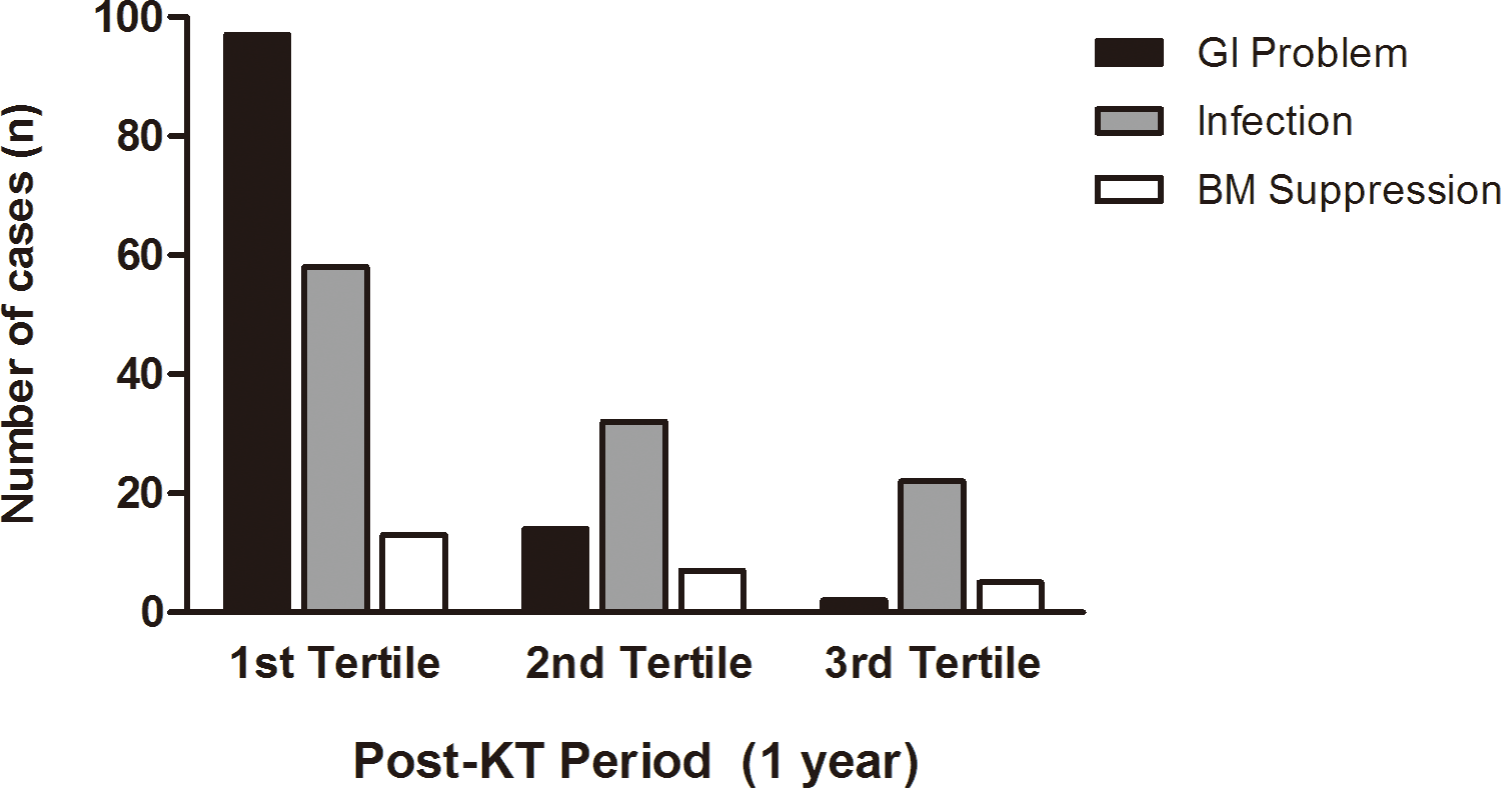
Three main reasons of immunosuppressant change according to post-transplant periods. The grouped vertical bar graph represents the frequency of the 3 most common causes of ISx according to post-KT periods evenly divided in to three sections. Black bars represent GI problem-induced ISx change, gray bars represent infection-induced ISx change, and white bars represent ISx change due to bone marrow suppression. All of three causes of ISx change were common in the first tertile period. The GI problem was the most common in the first tertile period, while infection became the main cause of the ISx change in the second and third tertile periods. Abbreviation: KT, kidney transplantation; GI, gastrointestinal; ISx, immunosuppressant.

Of the 379 ISx changes, the primary cause of the change was a GI problem (n = 113, 29.8%) followed by infection (n = 112, 29.6%), hematologic abnormality (n = 25, 6.6%), and simplification of the medication dose (n = 17, 4.5%). Acute rejection (n = 15, 4.0%), hair loss (n = 15, 4.0%) and nephrotoxicity (n = 9, 2.4%) also resulted in ISx changes (Fig 3). GI problems mostly developed in the first tertile after the KT, and were the leading cause of ISx changes during the early transplant period. On the other hand, infection occurred steadily during the first year of KT and became the most common cause of ISx change in the second and third tertile (Fig 4). Infection was reported 121 times in 87 recipients, with 112 (92.6%) of them causing the ISx change. Among the infectious complications, CMV infection was most common (n = 28, 25%), followed by BKV infection (n = 25, 22.3%).

### Changing pattern of immunosuppression

**Fig 5:**
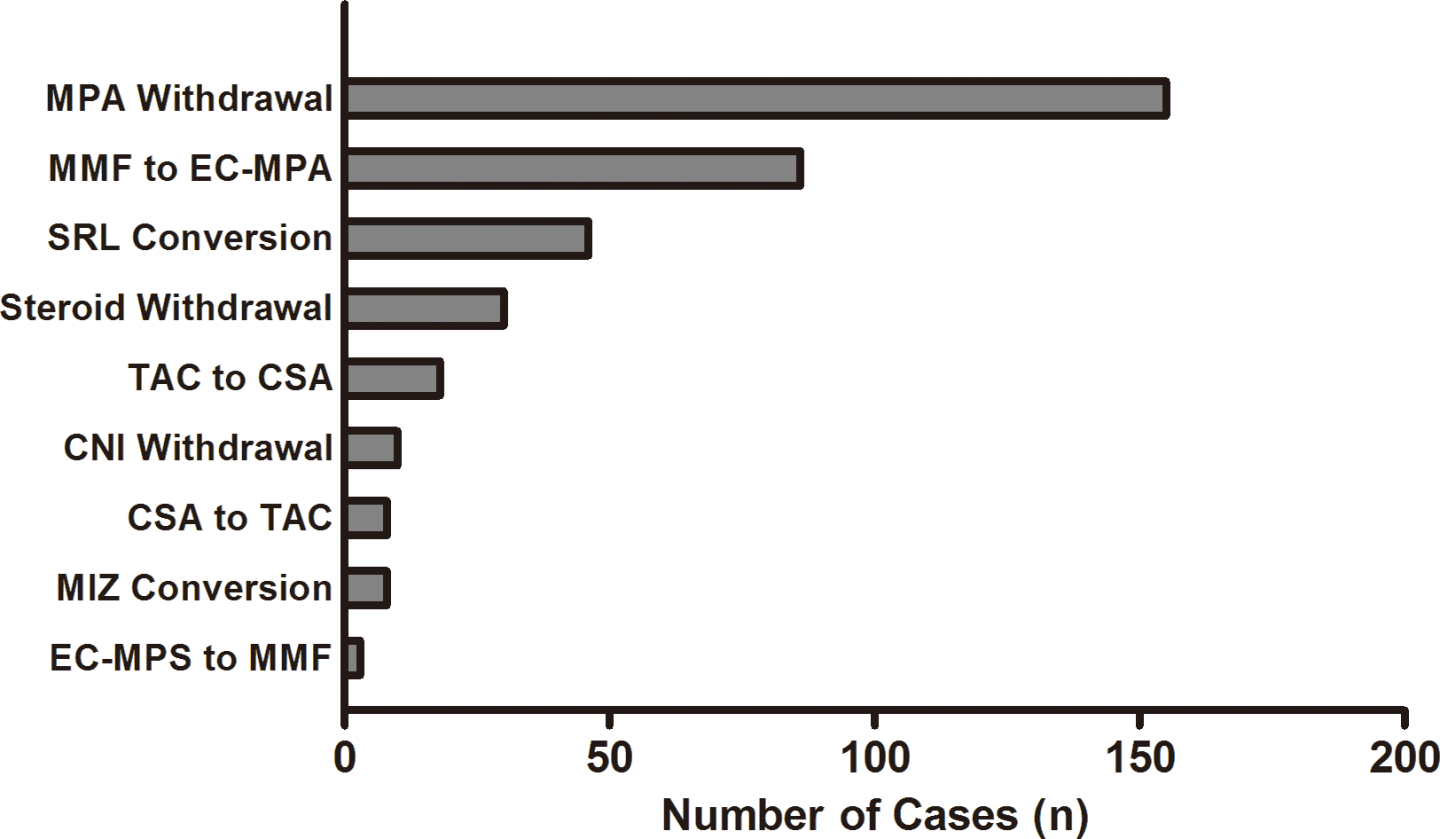
The changing pattern of immunosuppressants. The horizontal bar graph represents the changing pattern of ISx in order of the frequency. The total number of the changing cases was 379. MPA withdrawal was the most common changing pattern (155 occurrences, 40.9%), followed by the conversion from MMF to EC-MPS (86 occurrences, 22.7%). Abbreviation: MPA, mycophenolic acid; MMF, mycophenolate mofetil; EC-MPS, enteric-coated mycophenolate sodium; SRL, sirolimus; CNI, calcineurin inhibitor; TAC, tacrolimus; CSA, cyclosporine A; MIZ, mizoribine; ISx, immunosuppressant.

The most common pattern of the ISx change was MPA withdrawal (n = 155), followed by MMF to EC-MPS conversion (n = 86). Table 4 shows the cause of the MPA withdrawal. The clinicians opted to discontinue MPA in their patients due to various complications including infection, GI problems and bone marrow suppression. However, for the patients who complained of GI discomfort, many clinicians recommended switching from MMF to EC-MPS first. The SRL conversion was the third most common ISx pattern, observed in 46 cases. Among these, the conversion for better adherence to treatment with a once daily dosing occurred in 34.8% of patients (Table 4, Fig 5).

**Table 4:** Changing patterns of immunosuppression according to causes.

Cause of Change	The Most Common Pattern (n)	The 2^nd^ Most Common Pattern (n)
Gastrointestinal adverse event	MMF to EC-MPS (72)	MPA withdrawal (29)
Infection	MPA withdrawal (83)	SRL conversion (8)
Bone marrow suppression	MPA withdrawal (22)	Steroid withdrawal (4)
Dose of simplicity	SRL conversion (16)	CSA to TAC (1)
Acute rejection	SRL conversion (4)	CSA to TAC (3)
Hair loss	TAC to CSA (8)	MMF to EC-MPS (7)
nephrotoxicity	SRL conversion (7)	CSA to TAC (1)
DM complications	Steroid withdrawal (3)	TAC to CSA (2)
malignancy	SRL conversion (3)	MPA withdrawal (1)
cardiovascular toxicity	MPA withdrawal (2)	CNI withdrawal (1)
others	MPA withdrawal (17)	Steroid withdrawal (13)

Data account for the number (%) of immunosuppression changing pattern; total number = 396.

Abbreviation: GI, gastrointestinal; MMF, mycophenolic mofetil; EC-MPS, enteric-coated mycophenolate sodium; MPA, mycophenolic acid; SRL, sirolimus; CNI, calcineurin inhibitor; CSA, cyclosporine A; TAC, tacrolimus.

### Allograft function according to immunosuppressants change

There were two cases of graft failure and both occurred in patients who changed their ISx therapy. The one-year graft function was worse in the deceased-donor transplants than in the living-donor transplants (eGFR: 60.3 mL/min vs. 66.4 mL/min, p-value < 0.001), in patients using CSA than in those using TAC (eGFR: 57.8 mL/min vs. 64.8 mL/min, p-value = 0.005), worse in patients receiving CNI-based triple therapy than in those receiving other regimens (eGFR: 62.5 mL/min vs. 70.4 mL/min, p-value < 0.001), and worse in the ISx change group (eGFR: 59.7 mL/min vs. 66.8 mL/min, p-value < 0.001) (Table 5).

**Table 5:** Characteristics according to immunosuppression change.

		ISx Change		
		YES	NO	p-value
		(n = 251)	(n = 385)	
Age		46.7 ± 12.4	44.6 ± 12.7	0.043
Male, n (%)		137 (54.6)	240 (62.3)	0.058
DDKT, n (%)		105 (41.8)	147 (38.2)	0.363
Retransplantation, n (%)		20 (8.0)	25 (6.5)	0.528
Highly sensitized, n (%)		38 (15.1)	40 (10.4)	0.084
ABO incompatibility		38 (15.1)	47 (12.3)	0.341
Induction with ATG, n (%)		46 (18.4)	33 (8.9)	0.001
Acute rejection, n (%)		68 (27.1)	67 (17.4)	0.004
**GRAFT FAILURE, N (%)**		2 (0.8)	0 (0.0)	0.155
**EGFR** (mL/min/1.73m^2^)				
	At discharge after KT	70.0 ± 26.3	73.7 ± 24.0	0.066
	One year after KT	59.7 ± 23.2	66.8 ± 18.9	< 0.001
	∆ eGFR (1 year)	- 10.1 (23.1)	- 6.5 (22.6)	0.021

Continuous variables with normal distributions are presented with mean±S.D.

Continuous variables without normal distributions are presented with median (interquartile range).

**∆**eGFR represented the slope of eGFR between the time of discharge and 1-year after the transplantation.

Abbreviation: ISx, immunosuppressant; DM, diabetes mellitus; eGFR, estimated glomerular filtration rate; KT, kidney transplantation.

## Discussion

Several studies on ISx prescription trends have been reported [3–7]. Compared to other studies, this study focused on the actual prescription pattern for ISx changes, and the specific reasons behind each change. The results of our study clearly demonstrate that a considerable number of patients (39.5%) changed their ISx and most of those changes occurred due to drug-related events or medical events during the early transplant period. These findings suggest that clinicians should consider benefits and risk of choosing ISx in KT recipients.

Induction therapy was prescribed for most of the patients in this study (97.6%) with basiliximab in 85.2% of cases. This choice was appropriate according to the Kidney Disease: Improving Global Outcome (KDIGO) guideline in that an interleukin-2 receptor antagonist is recommended as the first-line induction therapy in all kidney transplant recipients [8]. The reason for preferential use of basiliximab, rather than ATG, may be based on its excellent efficacy and safety, as confirmed in large meta-analysis of randomized trials, as well as its lower risk of CMV infection and malignancy compared to that of ATG [8, 9]. On the other hand, the recent Scientific Registry of Transplant Recipients (SRTR) data in the USA showed a dominant position of ATG in the induction therapy [7]. The reason for the difference in proportion with the induction regimen between the two countries may be due to different donor types (proportion of deceased donor) and immunologic risk (proportion of sensitized patients).

An initial ISx therapy containing biologics induction plus low-dose TAC-based triple therapy has been broadly accepted based on the Symphony study [10, 11]. Most recipients in our study (81.4%) also received the initial maintenance therapy with a CNI-based triple regimen, and TAC was used seven times more frequently than CSA was (87.7% vs. 12.3%). Most centers favored the CNI-based triple therapy, but two centers initially chose either a steroid-free regimen (n = 72) or an MPA-free regimen (n = 40). These dual regimens comprised 17.6% of all patients; the immunological risk of patients receiving dual therapy was similar to the others. Taken together, most maintenance ISx therapies comprise a CNI-based triple regimen, but selection of ISx is also influenced by center practice.

After one year, the proportion of CNI-based triple therapy decreased by 14% (81.4% to 67.5%). On the other hand, the proportion of SRL-based regimens increased by 7.4% (0.3 to 7.7%), and that of steroid-free regimens increased by 1.9% (11.3 to 13.2%). The proportion of SRL-based regimens steadily increased, eventually leading to a decrease in CNI-based triple therapy. Interestingly, one-third of SRL conversions occurred due to the simplicity of a once daily dose (34.8%), and other causes were BKV infection (17.4%), nephrotoxicity (15.2%), and post-transplant malignancy (6.5%). In general, SRL is considered a replacement for CNIs due to its lower nephrotoxicity and anti-oncogenic effects. However, the results of our study suggested that the dose of simplicity for adherence was also important in determining the maintenance regimen.

Important finding of this study was that the most common cause of ISx change was GI problem and MPA was the most easily changed ISx. Our study revealed that clinicians chose two options (MMF to EC-MPS conversion or MPA withdrawal) when patients complained of GI discomforts, and favored converting to EC-MPS rather than MPA withdrawal (63.7% vs. 22.3%). The reason for higher rate of EC-MPS conversion may be related to previous reports indicating that EC-MPS conversion in KT recipients improved the GI tolerability of MPA treatment with maintaining immunosuppression [12–14]. Considering MMF-related GI discomforts and conversion rate of EC-MPS, it is also considerable to choose EC-MPS as an initial ISx.

Infection was the second most common cause of ISx change and common infectious causes were CMV disease (28 out of 112) and BKV infection (25 out of 112). For both virus infections, the most common pattern of ISx change was MPA withdrawal (71.4% for CMV and 44.0% for BKV). Compared to that for CMV disease, diverse options of ISx change exist with BKV infection including SRL conversion (28%), steroid withdrawal (12%) and conversion from TAC to CSA (8%). Treatment selection from these approaches may be dependent on the immunological risk of the recipients. However, a randomized control study is needed to identify the proper ISx change in BKV infection.

We also evaluated the time period of ISx change after the KT. In this study, more than half of the changes occurred during the first tertile period, and GI problem was common causes, while infection became the main cause of ISx change in the second and third tertile periods. Since ISx changes were mostly attributed to GI problems (97 out of 228) and CMV disease (21 out of 58 infectious causes) in the first tertile, both MPA withdrawal (92 out of 234) and MMF to EC-MPS conversion (80 out of 234) occurred with similar frequencies in patients during this period. In the second tertile, SRL conversion was the second most common pattern (24 out of 98) following MPA withdrawal, along with an increase in the prevalence of BKV infection (20% for the first tertile, 56% for the second tertile, 24% for the third tertile). MMF to EC-MPS conversion rarely occurred since second tertile. These findings suggest that ISx is changeable during early transplant period, and strategy for reducing risk factors (GI complication and BKV infection) is needed for maintaining initial ISx.

Because MPA was the most easily changed ISx, we further evaluated the MPA dose. Asian patients have a higher MPA exposure than do Caucasian or African American patients with a comparable MMF dose. For this reason, MMF dose in Asian transplant recipients may be reduced to 20% to 46% lower than in Caucasian or African American patients [15–18]. In this study, the average dose of both MMF and EC-MPS (1.4 g and 0.8 g, respectively) was lower than in Western countries. In this study, the mean dose of MMF was not statistically different between patients with side-effects and patients without side-effects (1382 mg vs. 1486 mg, p-value = 0.058). But the mean dose of EC-MPS was higher in patients with side-effects compared to patients without side-effects (925 mg vs. 788 mg, p-value = 0.002). The optimal dose of MMF or EC-MPS in Asian patients is still undetermined but the dose should be reduced considering the high prevalence of MPA-related complications in this study.

There was an unexpected finding of our study. CSA-using patients are generally expected to require a higher MPA dose than TAC-using patients due to a lower MPA exposure [19]. Here, the MPA dose in TAC-using patients was higher than in CSA-using patients. These opposite results may be explained by a higher immunological risk of patients using TAC than that of patients using CSA, and may suggest the need for a pharmacokinetic study in patients with complications, even in those with low-dose MPA.

There were some limitations in this study. Our study was conducted retrospectively for short-term duration of one year. Therefore, prospective and long-term follow-up study is needed to evaluate whether early change of ISx affects the long-term graft survival. In addition, we did not analyze the center effect. There is a report that the ISx choice was largely driven by center practice [4]. However, our study showed relatively uniform pattern of ISx protocol, and center effect was not strong to affect overall ISx prescription pattern. Taken together, our study provides a guidance regarding the ISx prescription to KT clinicians. Further researches including clinical trials and data analyses are necessary in order to achieve the better immunosuppressive therapy and allograft outcome.

In conclusion, current ISx with triple therapy is prone to be changed within one year in KT recipients. More detailed strategy for maintaining immunosuppression is needed in clinical practice.

## Supporting information

S1 File The individual data of kidney transplant recipients. (XLSX)

## References

[1] Lamb KE, Lodhi S, Meier-Kriesche HU. Long-term renal allograft survival in the United States: a critical reappraisal. American journal of transplantation: official journal of the American Society of Transplantation and the American Society of Transplant Surgeons. 2011;11(3):450–62. Epub 2010/10/27. doi: 10.1111/j.1600-6143.2010.03283.x . 20973913 10.1111/j.1600-6143.2010.03283.x

[2] Sis B, Mengel M, Haas M, Colvin RB, Halloran PF, Racusen LC, et al Banff '09 meeting report: antibody mediated graft deterioration and implementation of Banff working groups. American journal of transplantation: official journal of the American Society of Transplantation and the American Society of Transplant Surgeons. 2010;10(3):464–71. Epub 2010/02/04. doi: 10.1111/j.1600-6143.2009.02987.x . 20121738 10.1111/j.1600-6143.2009.02987.x

[3] Saemann MD, Sunder-Plassmann G. Maintenance immunosuppressive therapy in adult renal transplantation: a single center analysis. Transplant immunology. 2008;20(1–2):14–20. Epub 2008/09/23. doi: 10.1016/j.trim.2008.08.012 . 18804533 10.1016/j.trim.2008.08.012

[4] Axelrod DA, Naik AS, Schnitzler MA, Segev DL, Dharnidharka VR, Brennan DC, et al National Variation in Use of Immunosuppression for Kidney Transplantation: A Call for Evidence-Based Regimen Selection. American journal of transplantation: official journal of the American Society of Transplantation and the American Society of Transplant Surgeons. 2016;16(8):2453–62. Epub 2016/02/24. doi: 10.1111/ajt.13758 . 26901466 10.1111/ajt.13758 PMC5513703

[5] Ali AA, Al-Saedi AJ, Al-Mudhaffer AJ, Al-Taee KH. Five years renal transplantation data: Single-center experience from Iraq. Saudi journal of kidney diseases and transplantation: an official publication of the Saudi Center for Organ Transplantation, Saudi Arabia. 2016;27(2):341–7. Epub 2016/03/22. doi: 10.4103/1319-2442.178559 . 26997389 10.4103/1319-2442.178559

[6] Ponticelli C. Present and future of immunosuppressive therapy in kidney transplantation. Transplantation proceedings. 2011;43(6):2439–40. Epub 2011/08/16. doi: 10.1016/j.transproceed.2011.06.025 . 21839286 10.1016/j.transproceed.2011.06.025

[7] Hart A, Smith JM, Skeans MA, Gustafson SK, Stewart DE, Cherikh WS, et al Kidney. American journal of transplantation: official journal of the American Society of Transplantation and the American Society of Transplant Surgeons. 2016;16 Suppl 2:11–46. Epub 2016/01/13. doi: 10.1111/ajt.13666 . 26755262 10.1111/ajt.13666 PMC5541687

[8] Kasiske BL, Zeier MG, Chapman JR, Craig JC, Ekberg H, Garvey CA, et al KDIGO clinical practice guideline for the care of kidney transplant recipients: a summary. Kidney international. 2010;77(4):299–311. Epub 2009/10/23. doi: 10.1038/ki.2009.377 . 19847156 10.1038/ki.2009.377

[9] Webster AC, Playford EG, Higgins G, Chapman JR, Craig JC. Interleukin 2 receptor antagonists for renal transplant recipients: a meta-analysis of randomized trials. Transplantation. 2004;77(2):166–76. Epub 2004/01/27. doi: 10.1097/01.TP.0000109643.32659.C4 . 14742976 10.1097/01.TP.0000109643.32659.C4

[10] Ekberg H, Tedesco-Silva H, Demirbas A, Vitko S, Nashan B, Gurkan A, et al Reduced exposure to calcineurin inhibitors in renal transplantation. The New England journal of medicine. 2007;357(25):2562–75. Epub 2007/12/21. doi: 10.1056/NEJMoa067411 . 18094377 10.1056/NEJMoa067411

[11] Ekberg H, Bernasconi C, Tedesco-Silva H, Vitko S, Hugo C, Demirbas A, et al Calcineurin inhibitor minimization in the Symphony study: observational results 3 years after transplantation. American journal of transplantation: official journal of the American Society of Transplantation and the American Society of Transplant Surgeons. 2009;9(8):1876–85. Epub 2009/07/01. doi: 10.1111/j.1600-6143.2009.02726.x . 19563339 10.1111/j.1600-6143.2009.02726.x

[12] Salvadori M, Holzer H, de Mattos A, Sollinger H, Arns W, Oppenheimer F, et al Enteric-coated mycophenolate sodium is therapeutically equivalent to mycophenolate mofetil in de novo renal transplant patients. American journal of transplantation: official journal of the American Society of Transplantation and the American Society of Transplant Surgeons. 2004;4(2):231–6. Epub 2004/02/21. doi: 10.1046/j.1600-6143.2003.00337.x . 14974944 10.1046/j.1600-6143.2003.00337.x

[13] Budde K, Curtis J, Knoll G, Chan L, Neumayer HH, Seifu Y, et al Enteric-coated mycophenolate sodium can be safely administered in maintenance renal transplant patients: results of a 1-year study. American journal of transplantation: official journal of the American Society of Transplantation and the American Society of Transplant Surgeons. 2004;4(2):237–43. Epub 2004/02/21. doi: 10.1046/j.1600-6143.2003.00321.x . 14974945 10.1046/j.1600-6143.2003.00321.x

[14] Hwang HS, Hyoung BJ, Kim S, Oh HY, Kim YS, Kim JK, et al Improved gastrointestinal symptoms and quality of life after conversion from mycophenolate mofetil to enteric-coated mycophenolate sodium in renal transplant patients receiving tacrolimus. Journal of Korean medical science. 2010;25(12):1759–65. Epub 2010/12/18. doi: 10.3346/jkms.2010.25.12.1759 ; PubMed Central PMCID: PMCPMC2995230. 21165291 10.3346/jkms.2010.25.12.1759 PMC2995230

[15] Li P, Shuker N, Hesselink DA, van Schaik RH, Zhang X, van Gelder T. Do Asian renal transplant patients need another mycophenolate mofetil dose compared with Caucasian or African American patients? Transplant international: official journal of the European Society for Organ Transplantation. 2014;27(10):994–1004. Epub 2014/06/26. doi: 10.1111/tri.12382 . 24963914 10.1111/tri.12382

[16] Suhail SM, Vathsala A, Lou HX, Woo KT. Safety and efficacy of mycophenolate mofetil for prophylaxis in Asian renal transplant recipients. Transplantation proceedings. 2000;32(7):1757–8. Epub 2000/12/20. . 11119922 10.1016/s0041-1345(00)01388-9

[17] Tsang WK, Tong KL, Yeung S, Lee W, Chan HW. Efficacy and safety of mycophenolate mofetil in different dosages in Asian renal allograft recipients. Transplantation proceedings. 2000;32(7):1755–6. Epub 2000/12/20. . 11119921 10.1016/s0041-1345(00)01389-0

[18] Cho EK, Han DJ, Kim SC, Burckart GJ, Venkataramanan R, Oh JM. Pharmacokinetic study of mycophenolic acid in Korean kidney transplant patients. Journal of clinical pharmacology. 2004;44(7):743–50. Epub 2004/06/17. doi: 10.1177/0091270004266634 . 15199079 10.1177/0091270004266634

[19] Grinyo JM, Ekberg H, Mamelok RD, Oppenheimer F, Sanchez-Plumed J, Gentil MA, et al The pharmacokinetics of mycophenolate mofetil in renal transplant recipients receiving standard-dose or low-dose cyclosporine, low-dose tacrolimus or low-dose sirolimus: the Symphony pharmacokinetic substudy. Nephrology, dialysis, transplantation: official publication of the European Dialysis and Transplant Association—European Renal Association. 2009;24(7):2269–76. Epub 2009/04/10. doi: 10.1093/ndt/gfp162 . 19357111 10.1093/ndt/gfp162

